# Brain Magnetic Resonance Angiography of Subclavian Steal Syndrome

**DOI:** 10.31662/jmaj.2022-0129

**Published:** 2022-08-09

**Authors:** Tatsuya Tanaka, Kosuke Fukushima, Hirofumi Goto, Nobuaki Momozaki

**Affiliations:** 1 Department of Neurosurgery, International University of Health and Welfare, School of Medicine, Narita, Japan; 2 Department of Radiology, Imari Arita Kyouritsu Hospital, Arita, Japan; 3 Department of Neurology, Imari Arita Kyouritsu Hospital, Arita, Japan; 4 Department of Neurosurgery, Imari Arita Kyouritsu Hospital, Arita, Japan

**Keywords:** subclavian steal syndrome, time-of-flight magnetic resonance angiography, vertebral artery, subclavian artery, blood pressure

A 76-year-old man was hospitalized due to recurrent episodes of dizziness and a significant left-right blood pressure difference (right 106/59 mmHg, left 74/50 mmHg). Time-of-flight magnetic resonance angiography (TOF-MRA) revealed a gradual decrease in the signal intensity of the left proximal intracranial vertebral artery (VA) (Figure 1A arrow). Angiography showed an occlusion at the origin of the left subclavian artery (SA), reversed flow in the left VA, and identified subclavian steal syndrome (SSS) (Figure 2A). Percutaneous transluminal angioplasty with stenting for the proximal left SA occlusion was performed (Figure 2B and 2C). The postoperative course was uneventful, and the MRA revealed normalized signal intensities between bilateral VA (Figure 1B).

**Figure 1.**
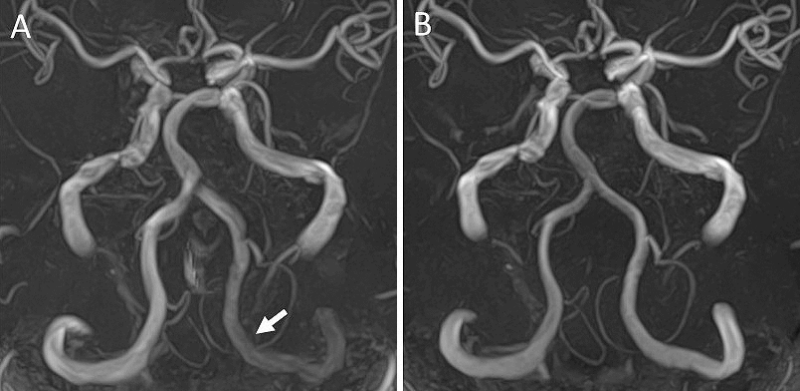
Time-of-flight magnetic resonance angiography (TOF-MRA) revealed a gradual decrease in the signal intensity of the left proximal intracranial vertebral artery (VA) (A). Postoperative TOF-MRA revealed normalized signal intensities between bilateral VA (B).

**Figure 2.**
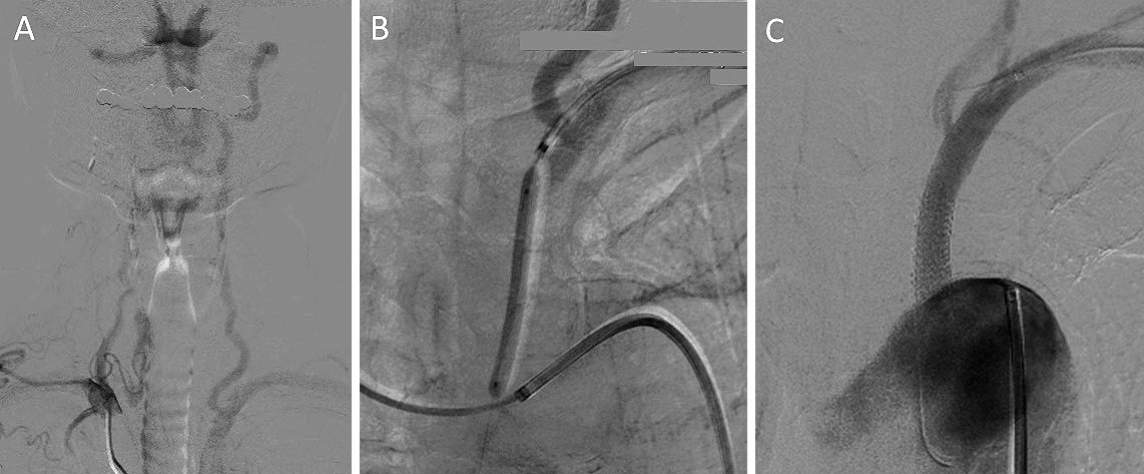
Angiography showed an occlusion at the origin of the left subclavian artery (SA), reversed flow in the left vertebral artery (VA), and identified subclavian steal syndrome (A). Percutaneous transluminal angioplasty with stenting for the proximal left SA occlusion was performed (B), which revealed normal flow in the left VA (C).

Blood flow in the affected VA of SSS is reversed and slow ^(1)^. Because of the use of radio frequency pulses, the MRA exhibited greater signal loss proximal to the VA. Our findings support the evaluation of the signal intensities of intracranial VA on the MRA to achieve an early diagnosis of SSS.

## Article Information

### Conflicts of Interest

None

### Author Contributions

TT wrote the first draft and managed all of the submission processes.

### Informed Consent

We have obtained informed consent for this case report.
